# The Short and Long-Term Effects of Pregnancy on Multiple Sclerosis and Experimental Autoimmune Encephalomyelitis

**DOI:** 10.3390/jcm7120494

**Published:** 2018-11-28

**Authors:** Pamela A McCombe

**Affiliations:** The University of Queensland, Centre for Clinical Research, Brisbane, QLD 4029, Australia; Pamela.McCombe@uq.edu.au; Tel.: +61-732-36-9960

**Keywords:** multiple sclerosis, pregnancy, experimental autoimmune encephalomyeltis, epigenetics

## Abstract

The role of pregnancy in multiple sclerosis (MS) is of importance because many patients with MS are young women in the childbearing age who require information to inform their reproductive decisions. Pregnancy is now well-known to be associated with fewer relapses of MS and reduced activity of autoimmune encephalomyelitis (EAE). However, in women with multiple sclerosis, this benefit is not always sufficient to protect against a rebound of disease activity if disease-modulating therapy is ceased for pregnancy. There is concern that use of assisted reproductive therapies can be associated with relapses of MS, but more data are required. It is thought that the beneficial effects of pregnancy are due to the pregnancy-associated changes in the maternal immune system. There is some evidence of this in human studies and studies of EAE. There is also evidence that having been pregnant leads to better long-term outcome of MS. The mechanism for this is not fully understood but it could result from epigenetic changes resulting from pregnancy or parenthood. Further studies of the mechanisms of the beneficial effects of pregnancy could provide information that might be used to produce new therapies.

## 1. Introduction

Multiple sclerosis (MS) is a common inflammatory disease of the central nervous system (CNS) [1,2]. The characteristic pathological features are inflammation and demyelination that occur in plaques, usually around a vein. There is also axonal degeneration, and as disease progresses there is microglial activation in so-called normal appearing white matter. The finding of lymphocytic infiltrates suggests that this is an immune mediated disease. Genetic studies show that the risk of MS is associated with genes of the immune system, most notably the genes of the human leukocyte antigen (HLA) region [3,4,5], but also other genes of the adaptive and innate immune systems. T and B lymphocytes and antibodies are thought to participate in MS pathology, and several CNS antigens have been proposed as the target of the immune attack [6,7]. In the peripheral blood of patients with MS, there is evidence of immune activation. This includes increased levels of T cells reactive with CNS antigens, and increased activated T cells [8,9]. Recently it has also been found that the gut microbiota influences the development of MS, possibly through immune effects [10], and that MS patients can have poor health for some years before presentation with neurological symptoms (this has been termed the “MS prodrome”) [11,12,13]. These findings of a role for the gut microbiota and as MS prodrome suggest that we need to reconsider the origins of MS.

Experimental autoimmune encephalomyelitis (EAE) has been used as a model of MS since the similarity between EAE and MS was noted in 1946 [14]. EAE can be induced by injection of CNS antigens and adjuvant or by adoptive transfer of T cells reactive to CNS antigens [15]. However, EAE differs from MS in needing to be induced, rather than occurring spontaneously. Usually EAE is induced with purified antigens, most commonly myelin basic protein (MBP), myelin proteolipid protein (PLP), and myelin oligodendrocyte glycoprotein (MOG), although a wide range of CNS antigens can be used [16]. The clinical course of EAE varies according to the inoculating antigen and the recipient strain (Table 1) [17,18,19,20,21,22,23,24,25].

Pregnancy involves physiological changes in the mother, including elevation of cardiac output, increased basal metabolic rate, increased lipid levels, and weight gain [27,28,29,30]. In pregnancy there are changes in the levels of hormones such as estriol, progesterone, prolactin, early pregnancy factor (EPF), alpha-fetoprotein [31], and leptin [32], and elevated levels of growth factors such as insulin-like growth factor (IGF) [33]. The physiological changes in pregnancy must be the net effect of all these hormones. After delivery, there is a rapid decline in the levels of pregnancy hormones and maternal physiology rapidly returns to normal, although during lactation the levels of prolactin and oxytocin are elevated [34]. It is thought that the changes in maternal physiology during pregnancy and lactation are mediated through the effects of pregnancy hormones [35]. These hormones have immunological and neuroprotective effects as summarized in Table 2.

The physiological changes in pregnancy include alterations in the maternal immune system. Immune changes in pregnancy tend to suppress the maternal immune system, and this is thought to be important in preventing rejection of the fetus, which contains foreign (paternal) antigens [44,45,46,47,48,49,50,51]. During pregnancy, there are increased Th2 responses and reduced Th1- and Th17-responses [35,52,53]. In pregnant women there are increased levels of circulating regulatory T cells (Treg) cells [54,55]. Other evidence of increased Treg activity in pregnancy comes from findings of increased foxp3 expression and increased functional suppression in pregnant and estrogen-treated rats [56]. The increased level of Treg cells in pregnancy is thought to be due to the effects on the immune system of estradiol [57]. The changes have been reported to include a shift to Th2 immune responses and a shift to greater humoral than cell mediated immunity [58]. B cells also show changes in pregnancy, with increases in regulatory B cells [59].

In the first part of the 20th century, MS was thought to be worse in pregnancy [60,61]. This idea was re-inforced by several influential case reports [62,63,64] and it was considered that pregnancy was a precipitant of MS [65]. However, a study by Tillman in 1950 cast doubt in the previously held view that pregnancy was not advisable in MS [61] and instead found that relapses of MS occurred after delivery. Further studies indicated that MS was better (fewer relapses) in pregnancy and relapses occurred post-partum. The issue of pregnancy and MS is currently of great interest and there have been a number of previous reviews of pregnancy in MS [66,67,68,69,70,71]. This review will provide an update on the data concerning the short and long-term effect of pregnancy in MS. It will also include a review of the effects of pregnancy in EAE. This has not been previously reviewed and could provide evidence of possible underlying mechanisms that are relevant to MS.

## 2. Multiple Sclerosis and EAE during Pregnancy

Current evidence from numerous studies demonstrates that there are fewer relapses of MS during pregnancy, especially in the later part of pregnancy [72,73,74,75,76,77,78]. A systematic review of 22 papers confirms the findings of reduced relapses in pregnancy [79]. However, it is worth noting that the risk of relapse in pregnancy is not zero, and recent studies in the era when patients are on immunomodulatory therapies have shown a rebound of disease in women who had previously been on active treatment with long washout periods [80]. There have also been reports of severe rebound of disease during pregnancy after withdrawal of fingolimod [81], and relapses, sometimes severe, in women who had been withdrawn from treatment with natalizumab [82,83,84,85].

The reduction in relapses during pregnancy is thought to be due to the immunological changes of pregnancy, as listed above. Some of the beneficial effects of pregnancy on MS could be due to effects of the pregnancy hormones on the nervous system, either in making neuronal tissue more resistant to damage or better able to repair damage [35,36,37].

There have been some studies of the immune changes in multiple sclerosis patients during pregnancy. There are changes in cytokines and T cells in pregnant compared to non-pregnant MS patients [86]. The frequency of interferon-γ producing T cells declined during pregnancy in women with MS [87]. A decline in interferon-γ was confirmed in a further study which also showed an increase in natural killer (NK) cells and a Th1–Th2 shift, but no changes in Treg cells [88]. A conflicting result was found in another study that showed an increase in CD4 + CD25^hi^ cells during pregnancy in patients with MS [55]. An increase in natural killer cells during pregnancy in MS was confirmed in a later study that also showed an increase in NK T cells [89]. There are changes in the expression of inflammation related genes during pregnancy in patients with multiple sclerosis [90].

There have been studies of the effects of pregnancy in EAE. EAE has a variable clinical course, according to the inoculating antigen, as shown in Table 1. The pathology of most types of EAE is characterized by microglial activation and infiltration with T cells and macrophages [16,91,92]. MBP-EAE [93] and PLP-EAE [94] can be transferred with lymphocytes, indicating that these cells are primarily responsible for these types of EAE. Disease is largely caused by Th17 cells [95,96] and is suppressed by regulatory T cells (Treg) [97]. In contrast, MOG-EAE is largely antibody mediated [98]. Acute EAE is a monophasic disease and animals do not have subsequent attacks, even if re-inoculated. The tolerance that occurs after acute EAE can be broken by mild immune suppression, suggesting that this is active tolerance [99,100]. Regulatory T cells play a role in the active control of EAE [97,101]. These features make EAE a useful model for evaluating the effects of pregnancy.

Pregnancy suppressed EAE in guinea pigs and rats [102]. Lewis rats inoculated with guinea pig spinal cord and adjuvant during the second or third weeks of pregnancy were protected against the development of disease, although rats inoculated in the first week had less protection [103]. Induction of MBP-EAE in Lewis rats during pregnancy also leads to reduced severity of disease [104]. SJL/J mice inoculated with PLP and adjuvant in the late part of pregnancy were protected against disease [105]. In PLP-induced EAE in SJL/J mice, immunization during pregnancy leads to a reduction in the incidence of EAE as well as a decrease in clinical severity, while immunization during the postpartum period leads to more severe disease [106]. In C57/BL6 mice, EAE induced with MOG 35–55 was less severe when mice were inoculated in late pregnancy [106]. In DA rats, EAE induced with bovine brain homogenate was less severe when disease was induced in pregnancy although there were relapses post-partum [107]. There have also been studies of the effects of becoming pregnant after the onset of EAE. In SJL mice inoculated with PLP 139–151 and in C57/BL6 mice inoculated with MOG 35–55, induction of pregnancy led to clinical improvement but the pathological lesions persisted [108].

There have been attempts to transfer the protective effects of pregnancy. In rats, offspring from rats with EAE in pregnancy show transient protection from EAE induced with GPSC, and rats that are suckled by mothers that have EAE in pregnancy also acquired protection [109]. Exosomes from pregnant and non-pregnant mice can suppress EAE. However, exosomes are more abundant in pregnancy and it is suggested that exosomes contribute to protection during pregnancy [110]. There is alteration of cytokine production in the spinal cord of pregnant rats with EAE compared to non-pregnant rats, and serum from pregnant rats suppressed lymphocyte proliferation in response to antigen and to phytohaemagglutinin, but not to concanvalin A, possibly indicating Treg activity [104].

There are no differences in lymphocyte proliferation or expression of activation markers when immunization occurred during pregnancy as compared with the non-pregnant controls [106]. Mice immunized during pregnancy produced less tumor necrosis factor-alpha (TNF-α) and interleukin 17 (IL-17), and showed an increased number of interleukin 10 (IL-10)-secreting cells within the CD11b+, CD11c+, CD19+, and CD4+/CD25+ Treg populations. No differences were noted in the production of interferon -gamma (IFN-γ), interleukin 2 (IL-2), interleukin 4 (IL-4), and interleukin 5 (IL-5). These results suggest that when an antigen is introduced during pregnancy, an immunoregulatory rather than an immunosuppressive or Th2 environment predominates. The glucocorticoid T cell receptor is necessary for the protective effect of pregnancy in MOG-EAE [111].

EAE can be suppressed by treatment with estrogen to produce levels similar to those found in pregnancy. This has been associated with changes in the gut microbiota [112] and with changes in regulatory B cells [113]. MS can also be suppressed by estrogen therapy [114]. This is a promising approach to try to replicate the beneficial effects of pregnancy, but it must be noted that many other hormones are elevated in pregnancy.

Taken together, the studies of MS and EAE indicate there are fewer relapses during pregnancy, increased relapses post-partum, alterations in the maternal immune system with reduced immune reactivity and increased regulatory cells.

## 3. Post-Partum Relapses

The studies that showed fewer relapses in pregnancy also showed an increased frequency of relapses in the post-partum period [72,73,74,75,79,115,116,117]. It is most likely that this effect is due to a loss of the immune suppressive effects of the pregnancy hormones. Another possibility is the effects of the hormones that are increased in lactation, but these have complex effects on the immune system (Table 1). Post-partum relapses are important because they lead to disability [118]. However, post-partum relapses do not occur in all patients, and there have been attempts to predict post-partum relapses using clinical and immunological approaches. The frequency of relapses before pregnancy is the best predictor of relapse after pregnancy [74,119,120]. One study of 298 full-term pregnancies found that breast feeding did not influence the frequency of relapses [120]. Relapses are nor predicted by epidural anaesthesia or caesarean section [121]. There have been studies suggesting that breast-feeding is protective against relapses [122]. However, later studies found that breast-feeding did not suppress disease [123] or influence the frequency of relapses [76,120]. One study suggests that post-partum relapses correlate with increased levels of IL-8 in the first trimester [124].

Some EAE studies provide information about the post-partum period. In PLP-induced EAE in SJL/J mice, mice immunized during the postpartum period exhibit more severe disease [106]. In DA rats, EAE induced with bovine brain homogenate was less severe when disease was induced in pregnancy although there were relapses post-partum [107].

There have been some clinical studies of methods to prevent post-partum relapses. In a retrospective analysis of study of patients who were followed in a database, 20 subjects who were given monthly intravenous methylpredisolone (1 g) for six months post-partum had a lower annualized relapse rate for the first three months post-partum than those who had no treatment [125]. There have been suggestions that intravenous immunoglobulin (IVIG) could be useful in preventing post-partum relapses [126,127,128] but a meta-analysis has failed to confirm this [129].

## 4. Relapses with ART Pregnancies

Increasing numbers of pregnancies now occur with assisted reproduction technology (ART). This generally involves the use of hormone therapy to induce ovulation or to assist in implantation. Such hormones can include gonadotrophin-releasing hormone (GnRH) agonists, follicle-stimulating hormone, luteinizing hormone, human chorionic gonadotrophin, and progesterone. In some patients, gonadotrophin releasing hormone antagonists are also used. When high doses are used, women can experience ovarian hyperstimulation syndrome. Women can be exposed to these hormones when ovulation is induced or when embryos are implanted. When considering whether ART influences MS relapses, it is important to note that when patients are trying to become pregnant, they often cease immunomodulatory therapy for MS, with a potential for return of disease activity.

There have been reports of the effect of ART on MS, as summarized in Table 3. The studies are small and some are retrospective. Relapses of multiple sclerosis have been reported to occur after pregnancies after ART [130,131,132]. A prospective study from Argentina showed the disease activity was increased 9-fold with use of luteinizing hormone releasing hormone (LHRH) agonists [133]. Two of the studies have suggested that relapses are more likely after treatment with LHRH agonists than antagonists [130,134]. In this respect, it is worth noting that GnRH has immune stimulatory effects [135] that could possibly lead to an increase in disease activity.

However, another group found an increased relapse rate in women treated with both GnRH agonists and GnRH antagonists, and suggested that relapses could be due to either to cessation of immune therapy, or to stress, which has been suggested to trigger relapses of MS, as well as to the possible effects of the hormones used in ART [131,132]. This needs further study to resolve these issues.

## 5. Use of Immunomodulatory Medications in Pregnancy

Whether to use of immunomodulatory therapies during pregnancy is a common clinical issue and a concern for women with MS. The rate of pregnancy in women with MS may be increasing, as shown in a study in the United States [136], so this topic is of importance. It is recommended that most of the medications are avoided during conception, pregnancy and breast-feeding. Furthermore, there needs to be a washout period to allow for the removal of the drug before conception is attempted. This avoidance of medications during pregnancy is because of suggestions of toxicity from animal studies but also because of a lack of evidence of safety of use in humans. A recent study showed that few women in the United States use immunomodulatory therapies during pregnancy or the puerperium [77].

However, although pregnancy is protective against relapses, the rate of relapse is not zero. Furthermore, in more recent studies there have been reports of relapses during pregnancy, possibly due to a rebound effect after ceasing effective therapy. This has led to a re-consideration of the possible use of medications during pregnancy, to protect the health of the mother. There are detailed reviews of these effects [70,71]. However, because of the risk of pregnancy and post-partum relapses, there have been attempts to find medications that can be used during pregnancy. The oral therapies and the lymphocyte-depleting therapies (alemtuzumab, ocrelizumab and cladribine) are not regarded as suitable for use during conception or pregnancy [70,71]. There are no significant issues with interferon beta. There is evidence that glatiramer acetate is safe in pregnancy and during breast feeding [137]. This is not unexpected because the molecule is rapidly degraded after absorption and is unlikely to reach the foetus in during pregnancy. It needs to be administered by injection so that it is not likely to be active if it were to enter the breast milk and be ingested by an infant.

Natalizumab is a monoclonal antibody, delivered by intravenous infusion. Antibodies can cross the placenta in the later part of pregnancy (after 28 weeks) and could affect the foetus. There have been reports of the use of natalizumab throughout pregnancy in patients with active disease [85,138,139,140]. The main adverse effects that have been noted in exposed infants are anaemia and thrombocytopenia in the newborn [141]. In the future, it is likely that MS patients, particularly those with active disease, will be offered some type of therapy during pregnancy.

Although the medications are not recommended during pregnancy, many pregnancies are unplanned, and exposures to disease modifying therapies have occurred. There are registries that are attempting to collect data about the outcomes of pregnancy exposure to disease modifying therapies. If a large number of exposed pregnancies were found to have the same outcomes as unexposed pregnancies, the recommendations regarding drug use in pregnancy could be re-considered.

## 6. Long Term Effects of Pregnancy on the Clinical Course of MS

The long-term effects of parity on MS is less certain. Some earlier studies suggested that parity is associated with better long-term outcome [69,142,143,144]. A retrospective study from Turkey suggested that parity had a beneficial effect on transition from relapsing to secondary progressive MS [145]. However, this could arise because women with more severe MS might decide not to have children or to have fewer children.

However, there is accumulating evidence that parity is beneficial. A prospective study examined the association between past pregnancy, offspring number, and risk of a first episode of demyelination [146]. The study demonstrated that higher parity was associated with reduced risk of a first episode of clinical demyelination and the results were consistent with a cumulative beneficial effect of pregnancy. In another study using the MSBase registry, where patients were matched according to clinical characteristics, pregnancy was 4.5 times more potent than first-line disease modifying therapies (interferon-beta, glatiramer acetate) in preventing long-term disability accrual, and any time spent pregnant (including induced and spontaneous abortions) was beneficial [147]. Other evidence of a benign effect of parity on MS is the findings that having been pregnant does not increase the risk of secondary progression of disease [148] and that women with children with different partners do not have greater risk of disability as might be expected to occur if the immune response to paternal genes was harmful [149].

For parity to have a long-term effect on MS, it would be expected that there would be permanent changes that persist after the woman is no longer pregnant and hormone levels have returned to normal. This could lead to accumulation of changes with successive pregnancies, due to the cumulative effects of pregnancy hormones, or to the effects of the experience of parenthood. One possible mechanism could be epigenetics, with changes in DNA methylation, leading to changes in gene expression. It is known that hormones such as estrogen can cause epigenetic changes [150,151]. It is also known that life experiences, such as stress and emotional well-being, can lead to epigenetic changes [152,153]. This could lead to cumulative epigenetic changes with parity and parenthood. If this is the mechanism by which prior pregnancies leads to better outcome of MS, it would be important to know which genes are involved. It can be speculated that such epigenetic changes could occur in immune-related processes or in processes to do with repair of the nervous system.

## 7. Effects of Pregnancy on Risk of Developing MS

Given the immune effects of pregnancy, it has been questioned whether pregnancy influences the development of MS. In a study from Denmark, parents of both sexes had a lower risk of MS than childless persons [154]. Because this effect was seen in both men and women, it was thought not to result of having been pregnant. It was thought that this could be due to reverse causality, as it is known that even before diagnosis of MS there are health changes [11,12,13]. Possibly those with poor health are less likely to decide to become parents than those with good health. This could lead to parenthood seeming to be protective from MS. However, one can speculate that parenthood could be protective in both sexes, possibly through epigenetic changes, similar to those proposed above for the effect of parity on the long-term outcome of MS.

## 8. Conclusions

The effects of MS on pregnancy and the effects of pregnancy on MS are common issues of concern for people with MS. Knowledge of this field is advancing and it is possible to provide information to address these concerns. It is now clear that pregnancy is not harmful, that there can be short term and possible long term beneficial effects. However, despite the beneficial effects of pregnancy there has been an understanding that in many women, treatment with pregnancy could be justified, and there is an increased emphasis on obtaining data that could provide more information about the risks and benefit of such treatment.

The mechanisms by which pregnancy reduces relapses and by which parity leads to a beneficial effect on outcome are still not fully understood. Further work in this area would be a great value, because understanding the mechanisms of the beneficial effects of pregnancy might lead to strategies to improve outcome of disease.

## Figures and Tables

**Table 1:** Common forms of experimental autoimmune encephalomyelitis (EAE).

Antigen	Species Studied	Clinical Course	Pathological Features	References
Guinea pig spinal cord	Rat	Monophasic	Inflammation and demyelination	[26]
Myelin basic protein	Lewis rat	Monophasic	Inflammation and demyelination, including nerve roots	[17]
Myelin proteolipid protein	Lewis rats, SJL mice	Chronic relapsing	Inflammation and demyelination	[19,20,21]
Myelin oligodendrocyte glycoprotein	Mice and rats	Chronic relapsing	Inflammation and demyelination, role for antibody	[22,23,24]

**Table 2:** Immune and neuroprotective effects of selected hormones of pregnancy and lactation.

Hormone	Timing of Increased Levels	Immune Effects	Reparative Effects	References
Estriol	Pregnancy	Anti-inflammatory	Neuroprotective, aids remyelination	[36,37]
Progesterone	Pregnancy	Anti-inflammatory	Neuroprotective	[38,39]
Prolactin	Pregnancy and lactation	Pro-inflammatory (controversial)	Possible role in neurogenesis	[40,41]
Oxytocin	Lactation	Complex effects	Neuroprotection	[42,43]

**Table 3:** Reports of relapses of multiple sclerosis (MS) with assisted reproduction technology (ART).

Study	Type of Study	No of Patients	No of ART Cycles	Relapses with GnRH Agonists	Relapses with GnRH Antagonists	Reference
Laplaud et al., 2006	Retrospective	6	10	5/6 treatments	0/4 treatments	[130]
Hellwig et al., 2008	Retrospective	6	14	3/9 treatments	2/5 treatments	[131]
Hellwig et al., 2009	Prospective/retrospective	23	78	Increased annualized relapse rate (ARR)	Increased ARR	[132]
Hellwig et al., 2009	Prospective group	10	14	2/8	1/4	[132]
Correale et al., 2012	Prospective	16	26	15/26	Not applicable	[133]
Michel et al., 2012	Retrospective	32	70	Increased ARR (48 treatments)	No increased ARR (19 treatments)	[134]
